# Human behaviours associated with dominance in elite amateur boxing bouts: A comparison of winners and losers under the Ten Point Must System

**DOI:** 10.1371/journal.pone.0188675

**Published:** 2017-12-29

**Authors:** Emily C. Dunn, Clare E. Humberstone, K. Fiona Iredale, David T. Martin, Anthony J. Blazevich

**Affiliations:** 1 Centre for Exercise and Sport Science Research, School of Medical and Health Science. Edith Cowan University, Joondalup, WA, Australia; 2 Australian Institute of Sport, Combat Centre, Canberra, ACT, Australia; University of Utah, UNITED STATES

## Abstract

Humans commonly ascertain physical dominance through non-lethal fighting by participating in combat sports. However, the behaviours that achieve fight dominance are not fully understood. Amateur boxing competition, which is judged using the subjective “Ten Point Must-System”, provides insight into fight dominance behaviours. Notational analysis was performed on 26 elite male competitors in a national boxing championship. Behavioural (guard-drop time; movement style [stepping/bouncing time]; clinch-time; interaction-time) and technical (total punches; punches landed [%Hit]; air punches [%Air]; defence) measures were recorded. Participants reported effort required (0–100%) and perceived effect of fatigue on their own performance (5-point Likert scale) following bouts. Differences between winners and losers, and changes across the duration of the bout were examined. Winners punched more accurately than losers (greater %Hit [33% vs. 23%] and lower %Air [17% vs. 27%]) but total punches, defence and interaction-time were similar. From rounds 1–2, clinch-time and guard drops increased whilst bouncing decreased. Perceived effect of fatigue increased throughout the bout while perceived effort increased only from rounds 2–3. %Hit and movement index together in regression analysis correctly classified 85% of bout outcomes, indicating that judges (subjectively) chose winning (dominant) boxers according to punch accuracy and style, rather than assertiveness (more punches thrown). Boxers appear to use tactical strategies throughout the bout to pace their effort and minimise fatigue (increased guard drops, reduced bouncing), but these did not influence perceived dominance or bout outcome. These results show that judges use several performance indicators not including the total number of successful punches thrown to assess fight dominance and superiority between fighters. These results provide valuable information as to how experienced fight observers subjectively rate superiority and dominance during one-on-one human fighting.

## Introduction

Fighting for dominance is a behaviour observed in many primates, including humans [1]. Dominating a rival in physical confrontation while peers observe has numerous social and physical advantages in the wild [2]; often a fight takes place in front of a crowd of peers, which helps the winner to consolidate their dominance within the group [3]. In modern human society, physical confrontation may be less common, however the instinct to assert dominance and promote hierarchical organisation is still entrenched [4, 5]. Combat sports provide an acceptable outlet for those desiring the challenges associated with physical confrontation and provide a vehicle for gaining insight into perceptions of dominance in humans.

In modern amateur boxing, male boxers compete in specified weight categories over three, 3-min rounds (each separated by 1-min of recovery) to determine a winner. Competitors aim to punch their opponent whilst avoiding their opponent’s punches. Until 2013, winners were decided by judges counting the number of clean blows landed to the target area at the front of the torso (on or above the line of the belt) and the front and side of the head [6], in 2013 the judging system was changed to the “Ten Point Must-System” (TPMS). After each round, judges award the winning boxer 10 points and the losing boxer between 6 and 9 points depending on their perception of the closeness of the contest. At the end of the contest, each judge awards the winner based on which boxer has the most points. Under the TPMS there are four criteria with which the judges assess the contest: 1) the number of quality blows on the target area, 2) domination of the bout by technical and tactical superiority, 3) competitiveness, and 4) lack of infringement of the rules [6]. In contrast to the previous scoring system, the TPMS deliberately incorporates a greater subjective component. Key words in the judging criteria indicative of the role of subjectivity are ‘superiority’, ‘dominance’ and ‘competitiveness’. In order to win, boxers must demonstrate superiority over their opponent across multiple criteria rather than simply landing more punches on the target area. In a recent study of the new rules, Davis and colleagues found that the accuracy of punches thrown, rather than the total number of punches landed, was higher in winners [7]. Such data suggest that subjective decisions regarding superiority, dominance and competitiveness are made using observational cues other than the total number of successful punches alone. The primary aim of this study therefore, was to determine which fight-related actions are more likely to be associated with winning under the TPMS through the examination of a wide range of technical, behavioural and perceptual variables during elite male amateur boxing bouts, with the assumption that this will provide insight into the cues used by humans (who regularly observe fights) to determine fight dominance.

The Cumulative Assessment Model (CAM) of fighting strategies integrates the metabolic cost and the cost of physical damage to theorise the outcome of contests between animals [8]. This theory declares that when a contestant suffers more fatigue than its rival it shall either retreat from or lose the contest [9], in both cases being less dominant. Given the strenuous nature of amateur boxing [10] fatigue may affect a boxer’s ability to perform in the ring [11]. We hypothesised that experiencing fatigue, or the demonstration of behaviours which indicate potential fatigue, during a competitive bout would affect the boxer’s behavioural and technical actions, which could in turn affect the judge’s perception of who was the dominant boxer. Therefore a secondary aim of this study was to monitor changes in technical and behavioural variables over the three rounds of elite boxing bouts, with added context being contributed by the athletes’ perceptions of effort and fatigue throughout, to determine if specific fatigue-related behaviours were associated with winners.

## Materials and methods

### Human participants

Twenty-six amateur boxers (mean age ± [SD] 22.2 ± 2.6 y) who competed in the Elite Male under 64 kg, under 69 kg and under 75 kg weight divisions at the 2015 Australian Boxing Championships participated in this study. All participants gave written informed consent before taking part in the study and were made aware they could withdraw their data at any time. The study was approved by the Human Research Ethics Committee at Edith Cowan University and all procedures were performed in accordance with the declaration of Helsinki.

### Data capture procedures

Participants competed in a boxing bout consisting of three 3-min rounds scored using the TPMS. Only bouts that lasted the full fight duration were selected for analysis. Video footage was captured at 50 frames/second from a video camera (AVCHD NXCAM, Sony Corporation, Tokyo, Japan) positioned at the ringside such that the whole bodies of both boxers were captured in the frame. Notational analysis of one bout per participant, from the first or second rounds of the tournament, was included in analysis (no boxer was analysed in more than one bout). Data were collected from 19 bouts, nine where both boxers were analysed and eight where only one boxer was analysed (due to their opponent having already been analysed as part of a previous bout). Notational analysis for each subject consisted of reviewing video footage between three and four times at one-quarter speed to tag and label specific techniques and behavioural patterns using the coding software (SportsCode Elite software; SportsTec, Hudl, Sydney, NSW, Australia). An experienced analyst conferred with elite boxing coaches to develop all variables recorded during notational analysis (Table 1). All bouts were analysed by the same experienced analyst who conferred with elite boxing coaches throughout a piloting phase. Three participants were analysed twice to determine the analyst’s intra-tester reliability (Pearson’s r = 0.98). The same three bouts were analysed by a second analyst (who was similarly trained) to determine the inter-tester reliability (Pearson’s r = 0.97) of the primary analyst. Only bouts analysed by the primary analyst were included in the statistical analysis. Perceptual variables were gathered via verbal surveys conducted within 30 min of the boxers leaving the ring after their bout. Athletes were asked “How much effort was required in round 1, 2, 3 and overall?” (recorded as a percentage) and “Do you think fatigue effected your performance in round 1, 2, 3 and overall” (recorded on a 5-point Likert scale). All perceptual ratings were collected by the same researcher.

**Table 1:** Description of variables collected.

Category	Variable	Unit	Description
Technical	Punches Thrown	Number	Total number of punches thrown
	Hit	Number	A punch that hits the target area
	Miss	Number	A punch that made contact with the opponent outside the target area
	Air	Number	A punch that failed to make contact with the opponent
	%Hit	%	The number of hits expressed as a percentage of total punches thrown
	%Miss	%	The number of misses expressed as a percentage of total punches thrown
	%Air	%	The number of air punches expressed as a percentage of total punches thrown
	Defensive Actions	Number	Number of all defensive techniques including arm, body and leg defence
Behavioural	Guard Drop	Seconds	Active lowering of the gloves, or holding a guard noticeably lower than when the fight commenced
	Bounce Time	Seconds	Time boxer spent with feet moving in an synchronised pattern
	Step Time	Seconds	Time boxer spent with feet move in an alternating pattern
	Movement Index	Ratio	Ratio of time spent bouncing to stepping
	Clinch Time	Seconds	Time while one or both boxers holding their opponent
	Interaction Time	Seconds	Time spend interacting with opponent (punching, defending etc.; excludes clinches)
Bout Descriptor	Referee Stoppage Time	Seconds	Time between referee calling “stop” and resuming the bout, does not include break calls
	Total Round Time	Seconds	Time between start and end bells
Perceptual	Effort Rating	%	Rating of how much effort was required during each round as a percentage of maximum effort
	Fatigue Rating	Rating 1–5	Rating on a 5-point Likert scale the extent to which boxers believed their performance was affected by fatigue in each round

### Statistical analysis

Data were analysed using IBM SPSS Statistics (version 19) and all data are expressed as mean ± SD. A 2-way multiple variable analysis of variance (MANOVA) with repeated measures was used to analyse groups of related variables (technical, behavioural, descriptor and perceptual) and interactions between rounds and groups. Alpha was set at p <0.05. Where significant effects were observed, a Tukey’s post-hoc test was used to identify where differences occurred. Cohen’s d effect sizes (ES) and 95% confidence intervals (CI) of the differences were calculated. Effect size magnitudes were classified using the scale advocated by Rhea (2004) for trained athletes in which <0.25, 0.25 − 0.5, 0.50 − 1.0 and >1.0 were termed trivial, small, moderate and large, respectively [12]. Binomial logistic regression analyses were performed on selected variables that represent accuracy (%Hit), volition (total punches thrown) and general movement style (movement index expressed as bounce: step ratio) in three different models: (1) %Hit only, (2) %Hit plus movement index and (3) %Hit plus movement index and total punches thrown.

## Results

### Comparison of winners and losers

Analysis showed a significant main effect (p = 0.043) of bout outcome (winners [n = 12] vs. losers [n = 14]) for technical variables (Table 2). Specifically, winners’ %Hit was significantly higher than losers in all rounds and showed large effects in rounds 1 (p <0.001; ES = 1.39; CI = 0.49 − 2.20) and 2 (p = 0.007; ES = 1.16; CI = 0.30 − 1.95) and moderate effects in round 3 (p = 0.007; ES = 0.87; CI = 0.04 − 1.65). %Air was significantly lower in winners than losers in round 2 (p = 0.005; ES = −1.08; CI = −1.87 − −0.23). Total punches thrown, total defensive actions, and %Miss were similar between winners and losers at each time point, with non-significant main effects being observed (p = 0.78, p = 0.87, p = 0.24, respectively).

**Table 2:** Technical variables for winner and losers for each round of boxing bouts.

		Round 1	Round 2	Round 3
Punches Thrown	Winners	75.8 ± 23.4	78.0 ± 29.4	75.7 ± 20.3
	Losers	78.6 ± 25.0	78.6 ± 28.8	80.7 ± 30.2
	All Boxers	77.3 ± 23.8	78.3 ± 28.5	78.4 ± 25.7
Hit	Winners	25.0 ± 10.5	26.3 ± 12.4	23.8 ± 6.2
	Losers	17.4 ± 8.2	19.9 ± 10.6	21.1 ± 9.3
	All Boxers	20.9 ± 9.9	22.9 ± 11.7	22.4 ± 8.0
Miss	Winners	37.3 ± 14.3	40.8 ± 12.3	39.8 ± 12.6
	Losers	43.2 ± 16.2	43.4 ± 16.8	44.9 ± 19.4
	All Boxers	40.5 ± 15.4	42.2 ± 14.7	42.6 ± 16.5
Air	Winners	13.6 ± 8.3	10.8 ± 7.9	12.1 ± 8.2
	Losers	18.0 ± 9.3	15.3 ± 6.5	14.6 ± 6.2
	All Boxers	16.0 ± 9.0	13.2 ± 7.4	13.5 ± 7.2
%Hit	Winners	33.1 ± 9.4	33.1 ± 6.9	32.0 ± 6.7
	Losers	21.6 ± 7.2 ‡	24.4 ± 7.9 ‡	26.2 ± 6.6 ‡
	All Boxers	27.0 ± 10.0	28.4 ± 8.5	28.9 ± 7.1
%Miss	Winners	49.2 ± 9.5	53.9 ± 7.2	52.8 ± 8.1
	Losers	54.6 ± 9.5	55.7 ± 8.3	54.6 ± 6.2
	All Boxers	52.1 ± 8.6	54.9 ± 7.7	53.8 ± 7.0
%Air	Winners	17.7 ± 9.0	13.0 ± 6.1	15.2 ± 8.7
	Losers	23.8 ± 10.3	19.9 ± 6.5 ‡	19.1 ± 7.4
	All Boxers	21.0 ± 10.1	16.7 ± 7.1	17.3 ± 8.1
Defensive Actions	Winners	31.8 ± 11.9	31.3 ± 12.6	29.4 ± 15.2
	Losers	30.6 ± 13.3	29.9 ± 12.4	29.6 ± 11.3
	All Boxers	31.2 ± 12.5	30.5 ± 12.3	29.5 ± 12.9

^‡^ = significantly different to winners at the same time point as determined by a 2-way MANOVA with repeated measures. Winners (n = 12), Losers (n = 14), All Boxers (n = 26).

Logistic regression models were significantly different from the null model. %Hit (*χ*^2^ (1) = 10.685, p <.001) explained 45% of the variance (Nagelkerke *R*^2^) and correctly classified 76.9% of winners and losers. When movement index was added to %Hit the model (*χ*^2^ (2) = 12.414, p = .002) explained 50.7% of variance and correctly classified 84.6% of bout results. Finally, when total punches was added to %Hit and movement index (*χ*^2^ (3) = 12.465, p = .006), 50.9% of variance was explained; however the prediction was slightly lower, correctly classifying only 80.8% of bout results.

There was no significant main effect (p = 0.42) between winners and losers for behavioural variables (Table 3). In some behavioural variables effect sizes indicated potential discrepancies between winners and losers but large confidence intervals made these outcomes unclear. Accordingly, moderate effect sizes for movement variables (movement index and step time and bounce time) suggested winners bounce more and step less than losers in all three rounds (movement index, round: 1 ES = −0.57; CI = −1.34 − 0.23; round 2: ES = −0.52; CI = −1.29 − 0.28; round 3: ES = −0.50; CI = −1.27 − 0.30; bounce time, round 1 ES = 0.68; CI = −0.13 − 1.45; step time, rounds: 1 ES = −0.69; CI = −1.46 − 0.12; round 3 ES = −0.59; CI = −1.36 − 0.21). Additionally moderate effect size (round: 1 ES = 0.62; CI = −0.19 − 1.39; round 2: ES = 0.63; CI = −0.18 − 1.40; round 3: ES = 0.53; CI = −0.27 − 1.29) suggests winners may drop their guard for longer durations in all three rounds compared to losers. There were no between-group effects for perceptual measures (p = 0.39; Table 3).

**Table 3:** Behavioural and perceptual variables for winners and losers and bout descriptor for each round of boxing bouts.

		Round 1	Round 2	Round 3
Guard drop (s)	Winners	28.8 ± 16.0	37.2 ± 22.8	39.5 ± 21.2
	Losers	20.0 ± 12.7	25.7 ± 13.2	29.5 ± 16.6
	All Boxers	24.1 ± 14.7	31.0 ± 18.8 *	34.1 ± 19.2 *
Step time (s)	Winners	56.0 ± 19.7	65.5 ± 23.8	56.0 ± 22.8
	Losers	70.8 ± 22.8	72.1 ± 28.9	69.1 ± 21.5
	All Boxers	64.0 ± 22.3	69.0 ± 26.4	63.0 ± 22.7
Bounce time (s)	Winners	66.9 ± 25.6	50.9 ± 27.6	49.4 ± 25.9
	Losers	49.0 ± 26.8	40.5 ± 27.9	39.7 ± 25.8
	All Boxers	57.3 ± 27.3	45.3 ± 27.7 *	44.2 ± 25.8 *
Movement index	Winners	1.27 ± 1.46	1.92 ± 1.57	1.71 ± 1.47
	Losers	2.20 ± 1.74	2.92 ± 2.18	2.63 ± 2.08
	All Boxers	1.77 ± 1.65	2.46 ± 1.95 *	2.20 ± 1.85
Effort rating (%)	Winners	65.8 ± 16.1	75.2 ± 11.6	85.7 ± 9.2
	Losers	79.2 ± 18.8	77.6 ± 22.1	90.5 ± 12.2
	All Boxers	73.0 ± 18.5	76.5 ± 17.7	88.3 ± 11.0 * †
Fatigue rating (1–5)	Winners	1.58 ± 0.67	2.08 ± 0.90	2.58 ± 1.56
	Losers	1.64 ± 0.84	2.29 ± 1.20	2.86 ± 1.66
	All Boxers	1.62 ± 0.75	2.19 ± 1.06 *	2.73 ± 1.59 * †
Referee stoppage time (s)	All Boxers	8.9 ± 9.0	18.5 ± 14.1 *	24.9 ± 16.8 * †
Total round time (s)	All Boxers	180.2 ± 1.5	.2 ± 10.0 *	185.3 ±9.0 *
Interaction time (s)	All Boxers	85.7 ± 15.8	93.2 ± 20.9	97.0 ± 17.7 *
Clinch time (s)	All Boxers	11.1 ± 10.0	17.1 ± 9.3 *	19.8 ± 11.7 *

* = significantly different (p<0.05) to round 1;

^†^ = significantly different to round 2 as determined by a 2-way MANOVA with repeated measures. Winners (n = 12), Losers (n = 14), All Boxers (n = 26).

### Changes in technique, behaviour and perception over time

Within-subject analysis of all participants (n = 26) showed that technical outcome measures remained consistent over the three rounds (no within-subject main effect) (Table 2). Analysis of behavioural variables showed a significant main effect (p = 0.001) over the three rounds of the contest. Specifically, absolute bounce time decreased from rounds 1–2 (p <0.001; ES = −0.42; CI = −0.63 − −0.22) and 1–3 (p <0.001; ES = −0.46; CI = −0.71 − −0.22), and movement index increased from round 1–2 (p = 0.017; ES = 0.40; CI = 0.09 − 0.72). However, absolute step time did not change significantly and only small effect sizes were observed over the rounds. Guard drop time increased significantly from rounds 1–2 (p = 0.012; ES = 0.46; CI = 0.11 − 0.80) and 1–3 (p = 0.002; ES = 0.66; CI = 0.27 − 1.06). Clinch time increased significantly, with moderate effects from rounds 1–2 (p = 0.004; ES = 0.57; CI = 0.22 − 0.93) and 1–3 (p <0.001; ES = 0.83; CI = 0.43 − 1.23). For perceptual measures, there was a significant within-subject main effect (p <0.001), with effort ratings increasing significantly from rounds 1–3 (p <0.001; ES = 0.80; CI = 0.50 − 1.10) and 2–3 (p <0.001; ES = 0.62; CI = 0.33 − 0.91). Fatigue ratings increased significantly at each time point, with moderate effect sizes for rounds 1–2 (p <0.001; ES = 0.74; CI = 0.41 − 1.08) and 2–3 (p = 0.01; ES = 0.69; CI = 0.20 − 1.19) and large effect sizes for rounds 1–3 (p <0.001; ES = 1.44; CI = 0.74 − 2.13).

### Interaction effects

There were no significant interaction effects between rounds and bout outcome (winners and losers) for the groups of variables analysed. Round-by-bout outcome interactions for technical, behavioural and perceptual variables were associated with p values of 0.620, 0.648 and 0.459, respectively.

## Discussion

**Fig 1:**
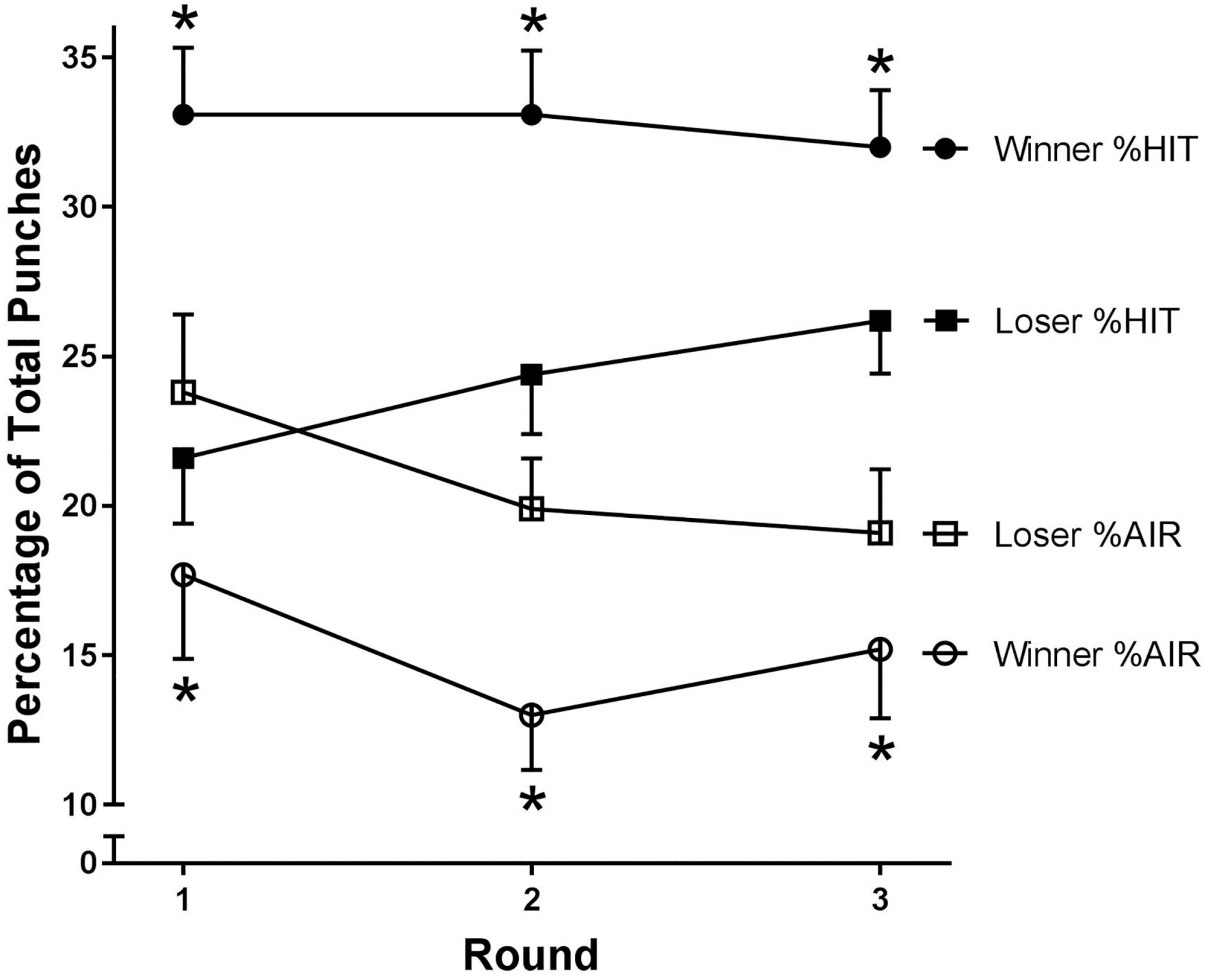
%Hit and %Air in winning and losing boxers over three rounds of tournament boxing. Winners are more accurate than losers, shown by significantly higher %Hit and significantly lower %Air compared to losers. Values expressed as mean ± SE; * significantly (p<0.05) different from losers as determined by a 2-way MANOVA with repeated measures.

The recent change to incorporate subjective judging criteria into amateur boxing challenges boxers to convince judges of fighting superiority and dominance. This provides a unique opportunity to study humans fighting for viewer-perceived dominance. The circumstance somewhat resembles fighting behaviour in humans and other primates in the animal kingdom and may provide insight into the cues used by humans to determine fight dominance (at least of experienced judges, who regularly observe fights). The results showed that winners had greater punch accuracy than losers, illustrated by a greater percentage of hits and lower percentages of air swings (Fig 1), but did not throw more punches in total. Thus, punch accuracy, rather than the total number of punches thrown, appears to be perceived as a key indication of dominance in well trained boxers. This finding is consistent with Davis and colleagues [7], who reported accuracy to be more favourable in winners than losers in a sample of elite male boxers competing at an international tournament. Interestingly, and also consistent with the findings of Davis and colleagues [7], neither the total number of punches thrown nor the absolute number of punches that were classified as hits, misses or air swings significantly differed between winners and losers. This finding suggests that having a high success rate is more favourable for victory than throwing and landing more punches than the opponent in total. Thus, the characteristics of winning boxers differs under the TPMS and previous ‘punch count’ system (where total number of successful punches characterised the winner [13]), and indicates that the judges’ perceptions of ‘superiority’, ‘dominance’ and ‘competitiveness’ are formed by more complex observations than the total number of successful punches thrown by a fighter.

Each instance in which a boxer hits the other is a complex encounter, but might be viewed as a function of both boxers’ skill levels. Hristovski and colleagues [14] demonstrated that boxers decided which punch to throw based on ‘reachability’, a skill that relies on visual cues and perceptions. Furthermore, Jackson and colleagues [15] reported that expertise level was related to the ability to use and detect deceptive actions in the collision sport of Rugby Union. This observation may be pertinent in boxing given the tactical use of feigning by combatants. It is possible that winners have developed these skills to a greater extent than losers, which allowed them to overcome the opponents’ defence systems (with the use of superior deception or feigning) to land the punches and avoid throwing air-swinging punches; however this hypothesis remains to be explicitly tested in subsequent studies. Judges’ perceptions of a boxer punching with efficiency (hitting often and air swinging infrequently) seem to be more positive than for boxers throwing a lot punches in total but with less efficiency (hitting and air swinging at similar rates). Indeed it is possible that as long as the judges believe the boxer looks good they may win the bout [7].

To explore the possibility that actions and behaviours other than punching accuracy could influence judge perception of dominance we selected and analysed behavioural variables such as dropping of the guard and style of movement around the ring (i.e. bouncing or stepping). This analysis revealed no statistical differences between winners and losers for the behaviours we monitored. When studying boxers who were fighting under the previous (punch count) system, when judge perception of how the boxers moved should not have influenced the bout outcome, winners were observed to display a greater number of vertical hip oscillations (VHO; defined as any visually identifiable vertical activity of the pelvis during stand and steps, which has been mainly attributed to bouncing) than losers [13, 16]. This result suggests that movement style might have had some influence on bout outcome. However, when studying boxers under the new TPMS, the same research group found no differences in VHO between winners and losers or changes throughout the bout [7]. In contrast, in the present study a tendency for winners to have a more vertical than translational movement style (i.e. more bouncing and less stepping) compared to winners was observed, with moderate effect sizes being calculated. Also, the logistic regression analyses revealed that the model with the best predictive outcome included information describing punch accuracy as well as movement styles; 84.6% of bout outcomes were correctly classified when the variables ‘%Hit’ and ‘movement index’ were included. Whether this is due to the movement style offering a technical advantage or whether it provides an aesthetic advantage and consequently contributes to a positive judge perception cannot be determined from the present data and should be investigated in future research (through interview of the judges, for example). However this outcome reinforces the hypothesis that judge perception might be influenced by more than just information relating to punching accuracy. The notion that movement style might influence perception of performance is not unique to boxing. Cormack and colleagues showed a fatigue-induced reduction in vertical acceleration during high speed running, which was associated with reduced coaches’ perception of the players’ performances in Australian Rules football players, irrespective of the players’ running rates (metres per minute and high-speed running metres per minute) during match play [17]. Such findings, in conjunction with the current results, indicate that humans may use general movement cues to make decisions regarding performance ability and the superiority of one athlete over another.

**Fig 2:**
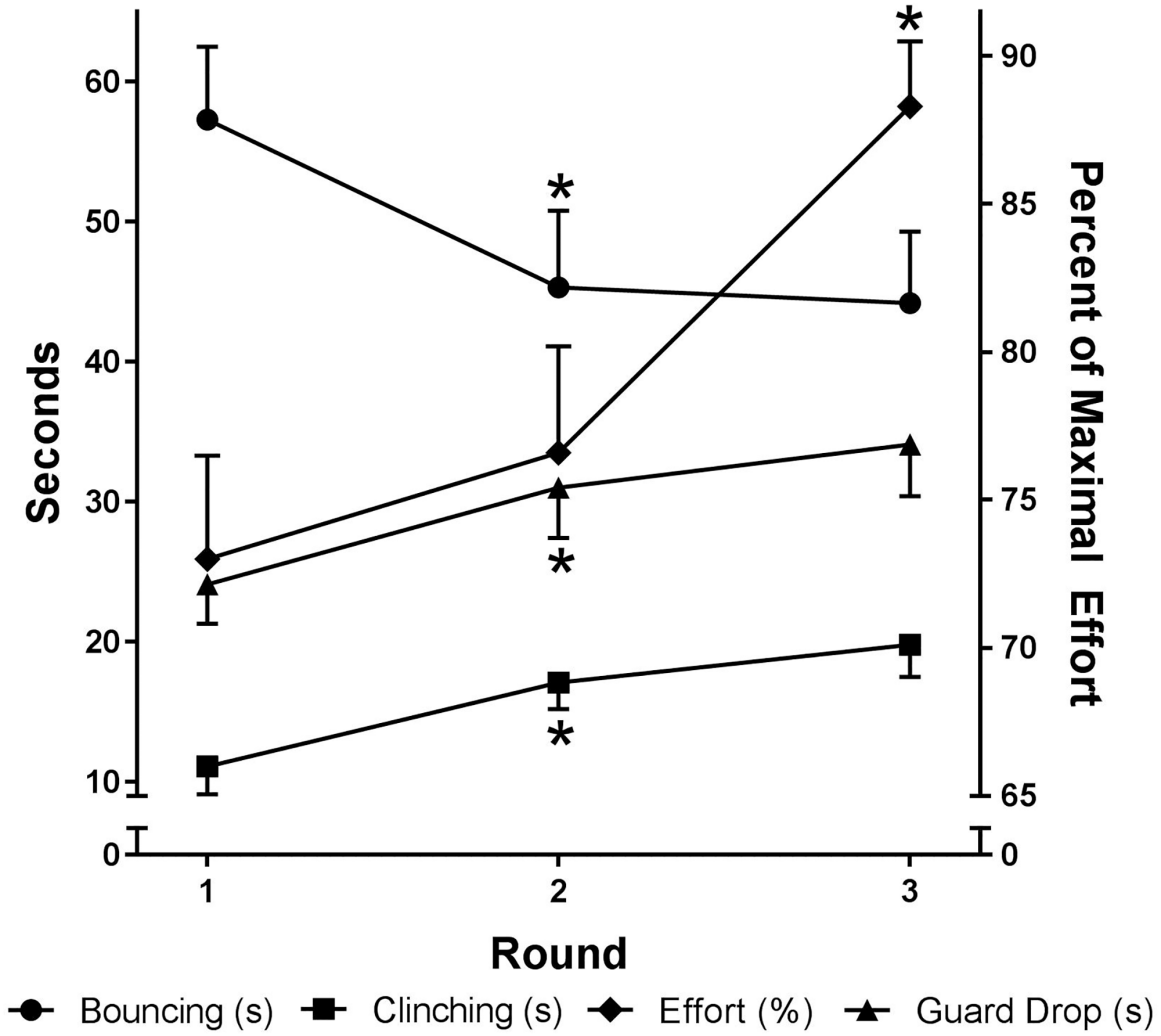
Behaviour (clinch time, guard drop time and bounce time) and perceived effort over three rounds of tournament boxing. Behaviour changes significantly from round 1–2 while perception of fatigue only from round 2–3, which may indicate pacing strategies have been used by boxers. Values expressed as mean ± SE; * significantly (p<0.05) different the previous round losers as determined by a 2-way MANOVA with repeated measures.

As boxing is a physically demanding sport (e.g. work: rest ratios as high as 19.3:1 [7]), fatigue can be linked to poor performance or behaviour change [18] and that fatigue may be the cause of defeat or the decision to flee in combative situations in animals [9]. We therefore also analysed changes in technical and behavioural variables over the duration of the match and included additional perceptual variables. The number of punches and defensive techniques used did not change throughout the bout. From this finding, one might conclude that the competition demands induced minimal fatigue. However, it is common for movement patterns to vary in order to maintain performance demands, a concept commonly referred to as pacing [18, 19] and defined as the regulation of exercise intensity with the intention to avoid early exhaustion while achieving a desired outcome [20]. The inclusion of perceptual data in the current study offers novel and unique insights that help us to better understand behavioural change during a boxing bout and provides information pertinent to pacing strategies, which might influence judge perception of dominance. Behavioural and perceptual variables, unlike technical variables, clearly fluctuated over the duration of the bout. Specifically, movement style, guard drops and clinching all changed from rounds 1–2 but not 2–3. The perceptual variables, on the other hand, followed a different pattern. Boxers’ perception of effort increased significantly only from rounds 2–3 while their perceived effect of the fatigue on their performance increased in all rounds (from rounds 1–2 and 2–3; Fig 2). This decoupling of fatigue rating, perceived effort and behaviour suggests that pacing strategies may be used at various stages of a boxing bout to mitigate the effect of fatigue. Furthermore, while increasing significantly, the extent to which boxers believe that their performance was affected by fatigue only reached moderate levels at the conclusion of the bout, which may have been because boxers effectively pace their efforts throughout the bout. Moreover, knowledge of the exercise end point might have also caused the late increase in effort required [21]; similar to the end spurt seen in most pacing profiles [22]. These findings indicate that movement patterns and behaviour such as guard dropping and clinching could be altered as a pacing strategy to avoid fatigue over the duration of an amateur boxing bout.

The behavioural concepts measured have been referred to in existing literature, although the pertinence to fatigue and pacing as not been explored before. Increases in guard drop are consistent with previous literature [13, 16, 23] and, if not specifically used for tactical purposes (e.g. to change the behaviours seen by the opponent), could be considered to be behaviours adopted to gain brief periods of rest for the smaller muscle groups of the upper body. Allen and Westerblad [24] suggested that a rest as short as a few seconds can be sufficient for the partial, rapid recovery of working muscle. The increase in clinching time (round 1–2) in all boxers might be a preferable behaviour in the later stages of a bout (when fatigue might become apparent) in order to avoid the opposing boxers optimal striking zone (i.e. clinching is a safety mechanism). However, clinching could also be used as a means to draw a stoppage and gain a brief rest. Collectively, altered movement style, guard and clinching behaviours could be used by boxers to moderate their exertion over the bout to maintain the number of punches and defensive actions used. Viewed in conjunction with these behaviour changes, the inclusion of perceptual data in the current study allows us to expand on this idea by providing extra information pertinent to pacing strategies.

As highlighted previously there are clear behavioural and perceptual changes in all boxers throughout the bout and clear differences in punch accuracy between winners and losers. We hypothesised that experiencing fatigue, or the demonstration of behaviours which indicate potential fatigue, could affect the judge’s perception of who was the dominant boxer. In that case one might expect the behavioural changes over the course of the bout to become more pronounced in winners rather than losers. However, in the present study there were no significant interaction effects between time and the outcome of the bout. This indicated that winners were consistently more accurate than losers, but that behaviour change followed the same patterns in all boxers regardless of winning or losing the bout. Our findings are consistent with previous literature in that punch accuracy is the most important factor to ensure victory, although we also acknowledge that movement style may have some effect on the judges’ perceptions or have a technical advantage for boxers. However to be successful in a boxing bout these characteristics must hold true over the duration of the bout.

## Conclusion

In conclusion, under the current subjective TPMS scoring system in amateur boxing, punch accuracy appears to be more important than the total number of punches thrown as winners had greater punch accuracy than losers (greater percentage of hits and lower percentages of air swings) but total punches thrown had no detectable effect on bout outcome. It is possible that winners have superior skill sets and were able to overcome their opponent’s defence system to land accurate punches, and that this was more important to ‘subjective dominance’ than overall assertiveness or volition (i.e. total punches thrown). Logistic regression analysis indicated that high punch hit percentage in conjunction with a vertical movement style was perceived by judges to indicate superiority. These data suggest that judges may not only take note of the punches that hit the opponent, but also use general movement patterns such as how the boxer moves around the ring, to decide which fighter is superior to the other. It is interesting that although boxers appeared to pace their effort to minimise the effects of fatigue by intermittently dropping their guard and using a translational movement style, there was no clear interaction between how these general movement patterns and behaviours change and the outcome of the bout. Regardless of win or loss, our analysis of boxers’ behaviours showed clear changes across the duration of the bout and indicated that winning and losing boxers adopt similar pacing strategies. While fatigue and pacing may be considered in the assessment of fighting dominance, it appears that in this case all fighters (winners and losers) are affected similarly throughout the bout and the changes observed have no effect the judge’s perception of who was the dominant fighter.
